# Retrieved Unicompartmental Implants with Full PE Tibial Components: The Effects of Knee Alignment and Polyethylene Thickness on Creep and Wear

**DOI:** 10.2174/1874325000802010051

**Published:** 2008-04-11

**Authors:** Ph Hernigou, A Poignard, P Filippini, S Zilber

**Affiliations:** University Paris XII, Hôpital Henri Mondor, 94010 Creteil, France

## Abstract

Creep and true wear of polyethylene are difficult to evaluate on radiographs of knee arthroplasties and for this reason the true rate of polyethylene wear *in vivo* after unicompartmental arthroplasty is not well known. This study evaluated the creep and true wear in fifty-five medial retrieved unicompartmental implants that had a flat articular surface at the time of implantation.

All the full polyethylene tibial components had the same design and were retrieved from eleven to 224 months (mean 152 months) after their implantation. The postoperative varus deformity had been measured on weight-bearing radiographs of the whole limb (hip-knee-ankle angle). The retrieved implants were placed in a coordinate measuring machine and the coordinates of a grid of points were obtained. Using this system, a three dimensional scaled image of the implant could be created and was used to calculate the total penetration of the femoral condyle due to true wear and creep.

Total linear penetration rates ranged from 0.18 to 2.6 millimeters per year (mean 0.25 millimeters per year). Linear penetration rates due to true wear ranged from 0.08 to 1.4 millimeters per year (mean 0.13 millimeter per year), and penetration due to creep ranged from 0.07 to 1.9 millimeters per year (mean 0.12 millimeters per year).

The linear and volumetric penetration rates of the femoral condyle due to true wear were negatively correlated with the duration of implantation. The linear penetration rate due to creep was higher in the first two years after the implantation compared to the subsequent years. Using multiple linear regression analyses to remove the confounding effects of age, weight, gender and thickness of the implant, we found that an increase of the postoperative varus deformity was due to an increase of creep (p = 0.03) but not with an increase of true wear (p = 0.25). Thinner implants were due to an increase of creep (p = 0.02) but not with an increase of true wear (p = 0.34). Increase in age was in relation with decrease of wear (p = 0.02) and increase of weight with increase in creep (p = 0.03).

Plastic deformation had a high influence on the penetration rate of the femoral condyle in full polyethylene implants. There is a risk of an increased penetration and a decrease of the remaining thickness of the tibial plateau when the implant is too thin, the knee mal-aligned and the patient heavy - each of these factors increasing the creep deformation. In conclusion, our study suggests that surgeons using the Lotus Mk I unicompartmental knee replacement for medial tibiofemoral arthritis should beware of the overweight patient (>90kg) with a tibial implant of less than 9 mm. There is a risk of increased penetration and decreased thickness of the tibial implant when it is too thin, the knee malaligned, and the patient heavy. All these factors increase creep deformation.

The linear and volumetric wear rates of polyethylene hip components have been documented extensively and it has been demonstrated that volumetric wear is influenced by many factors including activity head diameter, surface roughness and so on. Polyethylene wear mechanisms have been documented extensively. The hip represents a congruent articulation and creep of the polyethylene is not very great as regards true wear which occurs mainly by surface adhesion or surface abrasion. With flat or incongruent tibial inserts in knee arthroplasty [1], plastic deformation of the polyethylene (creep) may be great and influence volumetric wear measurement [2].

This study evaluated separately the amounts of creep and true wear in polyethylene tibial compoments that had a flat articular surface at the time of implantation and that were retrieved for loosening or for another cause. The postoperative alignment of these knees was measured on standing postoperative radiographs of the whole limb as the hip-knee-ankle angle. We have therefore specifically assessed the effects of the postoperative alignment of the knee and the effects of polyethylene thickness on the creep and wear of tibial polyethylene inserts retrieved at the time of revision surgery. To remove a possible confounding effect of wear by absence of anterior cruciate ligament, only knees with a present anterior cruciate ligament at the time of the operative when revision were included in this study.

## MATERIAL AND METHODS

### 1 Series

Fifty-five medial polyethylene tibial components of the same design were retrieved from forty-eight patients eleven to 224 months (mean 152 months) after their implantation. All the tibial and femoral implants had been inserted with cement. The model was the lotus Mark 1 developed by the GUEPAR group, with a flat polyethylene tibial component and a resurfacing femoral component. The implants had the same design and were implanted with the same technique. All the patients had the same standard follow-up, as well as the same standing X-rays. These fifty-five knees had present anterior and posterior cruciate ligaments at the time of implantation and at the time of revision. A medial parapatellar approach was used. No ligamentous release was undertaken. With femoral and tibial components in place, movement of the knee was tested. The thickness of the tibial implant was chosen so that the lateral and medial capsulo ligamentous structures were adequately balanced and under physiological tension. These polyethylene implants were sterilized with ethylene oxide gas. The clinical and radiographic status was available for each knee and each patient: the patient’s age, gender and weight at the time of the operation together with the original thickness of the components before implantation. Only knees with the anterior cruciate ligament present at revision were included in the study. The duration in situ was recorded and the postoperative deformity of the limb measured by the hip-knee-ankle angle.

The postoperative varus deformity was measured during the first six months on weight-bearing radiographs of the whole limb (hip-knee-ankle angle); all the subjects were asked to stand with the anterior part of the knees facing forward and the posterior part of the knees facing the film. A cassette holding long radiographs was placed behind the patient. Both lower extremities were included in one radiograph. The weight-bearing anteroposterior radiograph of the entire lower limb was made with the X-ray beam centered at the knees at a distance of three meters and the foot orientated in the direction of the X-ray beam. The hip-knee-ankle angle was formed by the angle between the line joining the center of the femoral head to the center of the knee and the line joining the center of the knee to the center of the ankle. Normally these axes form a straight line (180 degrees). Therefore in presence of a varus deformity the angle is less than 180 degrees. The mean post-operative hip-knee-ankle angle was 172 degrees (range 157 to 188 degrees) for these fifty-five implants that were retrieved eleven to 224 months after their implantation. As specified in the manufacturer packaging, the implant thickness was six, nine, twelve or fifteen millimeters. The sterilization had been done with ethylene oxide gas.

Thirteen of these components were retrieved before loosening. They included six implants that were retrieved during revision surgery because of wear of the opposite compartment, four because of involvement of the patello-femoral joint and three that were retrieved due to infection. The remaining implants were retrieved for prosthetic loosening. All the fifty-five knees still had their anterior cruciate ligament present at the time of implantation. These implants were obtained from different orthopedic centers, most of them (forty-two) coming from only two centers. The mean duration of the components in situ was 152 months (range, eleven to 224 months).

### 2 Study of Wear on Retrieved Components

**Fig. (1):**
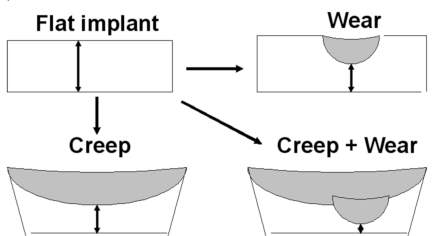
Drawings representing the method used to calculate the linear penetration related to true wear and the decrease of thickness of the implant due to creep. The implant was flat before implantation. The difference between the initial thickness and the remaining thickness at revision was considered to be the sum of creep and wear.

The remaining thickness of the implant was measured at the worn part (at the point of the greatest penetration of the femoral implant in the component). It was noted that some retrieved implants had a slightly curved surface probably due to a creep phenomenon. To separate the decrease in thickness due to wear from that due to deformation (creep) of the flat component, we first calculated the contributions of both creep and wear and then the contribution of wear only (Fig. **1**).

**Fig. (2):**
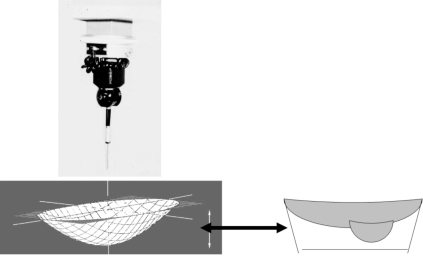
The change of volume at the surface of the implant was considered to be due to both creep and wear and was calculated with a coordinate measuring machine.

We used a coordinate measuring machine to determine by direct measurements the contribution due both to wear penetration and to deformation of the polyethylene (Fig. **2**). A high precision dial gauge (Mitutoyo/MTI) graduated to 0.003 millimeter and displaced automatically at 0.1 millimeter on parallel and equidistant lines was used to measure the depth of the worn area. The reference surface was considered to be a plane because the design of the implant was flat polyethylene. The flatness of never-implanted tibial implants was assessed with the high precision dial gauche (deviation less than 0.05 millimeters). The retrieved tibial components were placed in the coordinate measuring machine and the coordinates of a grid of points on the articular surface of the tibial component were then measured. These coordinate points were then used to define the area of the inner surface of the retrieved tibial component. Using this system, a three-dimensional scaled image of the entire polyethylene implant could be created on the computer and was used to calculate the change in volume due to penetration of the femoral condyle (true wear) and due to creep.

**Fig. (3):**
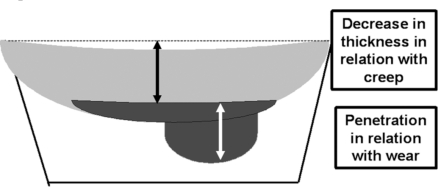
Creep was calculated by the computer by subtraction of the measured wear from the virtual image of the PE implant. This allowed to obtain the penetration due to wear and the decrease in thickness with creep.

To separate plastic deformation from true wear, the weight of true wear was calculated by weighing the tibial components and comparing the results with non implanted components of the same size. Density of the polyethylene was calculated by measuring volume and weighing several components before implantation. To correct for fluid absorption in the polyethylene, components were soaked in serum during several months. The weight was measured every month until a steady state that occurred at one year. Creep was calculated by the computer by subtraction of the wear measured by weighing from the virtual image of the polyethylene implant (Fig. **3**). The difference between the initial thickness of the polyethylene and the penetration due to wear was considered to be due to the deformation of the polyethylene considering that it was perfectly flat before implantation. The true wear was measured as the linear and volumetric penetration rates per year in the component and the creep phenomenon only as a linear rate of decrease thickness of the implant.

### 3 Statistical Analysis

The outcome measurements in the analysis were the creep deformation, the linear wear, and the volumetric wear rate measured by weighing the implants. Multiple regression analysis was used to determine differences in alignment of the implants among measurements of wear rates, while adjusting for any confounding effects of other variables. Separate regression analyses were performed. The dependant variables in the analysis were the postoperative hip-knee-ankle angle and the thickness of the implant. Independent variables included age, weight, gender, the revision status, and the outcome measurements of wear and creep. Weight was chosen as variable rather than body mass index because the polyethylene was flat and the contact on the polyethylene as a point, and so independent of the size of the implant and independent of the size of the patient. We also examined the effects of age, weight, duration of implantation on the influence of creep and true wear with the Spearmann correlation test. Statistical significance was set as p < 0.05.

## RESULTS

### 1 Decrease of the thickness of the implant due to true wear measured by weighing the polyethylene:

The amount of volumetric wear ranged from 113 to 2684 cubic millimeters (mean 709 cubic millimeters). The amount of decrease in thickness of the polyethylene implant due to wear was average 1.6 millimeters (range 0.8 to 4.1 millimeters).

**Fig. (4):**
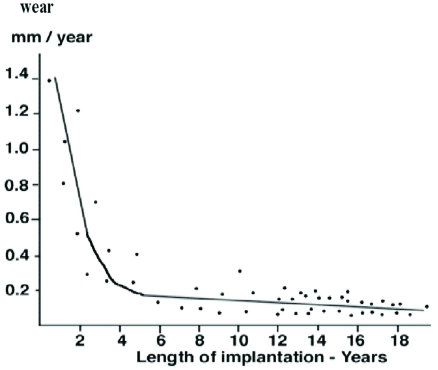
The linear polyethylene wear rate compared to the duration of implantation.

**Fig. (5):**
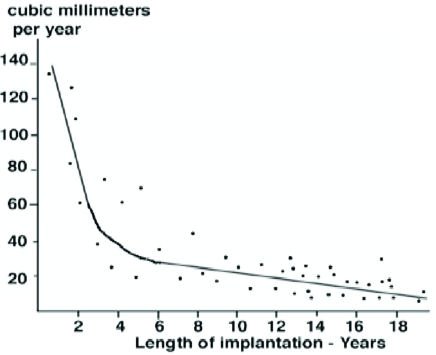
The volumetric polyethylene wear rates compared to the duration of implantation.

Linear and volumetric penetration due to true wear ranged respectively from 0.08 to 1.4 millimeter per year (mean 0.13 millimeter per year) and from eleven to 135 cubic millimeters per year (mean fifty-six cubic millimeters per year). Linear and volumetric penetration rates due to true wear were negatively correlated with the duration of implantation (Figs. **4**,**5**). When linear and volumetric rates of wear were plotted against duration of implantation, there proved to be a higher wear rate in the first years of implantation compared to subsequent years. After the fourth year of implantation the linear and volumetric wear rates remained fairly constant (and even slightly decreased). Since the tibial implant was flat, the higher wear rates during the first fourth years were probably due to the creation of a small dish in the polyethylene by the femoral implant as represented in the Fig. (**4**).

We found no statistically significant relationship between the annual wear rate (linear or volumetric) and the weight or gender (p > 0.05). Using the Spearman test, these linear and volumetric wear rates showed a negative correlation (p < 005) with patient age (Rs = 0.21; Rs = 0.31). The patients with the higher annual rates of wear (greater than 0.11 millimeter per year) after the fourth year of implantation were significantly (p = 0.02) younger (mean, 64 years; 95 per cent confidence interval 59.7 to 69.3) at the time of implantation than those (mean, 71 years; 95 per cent confidence interval 65.4 to 76.6) with the lower rates (less than 0.11 millimeters per year) after the fourth year of implantation.

The amount of true wear determined by weighing was not significantly (p = 0.34) greater in the thinner (≤ 9 millimeters) polyethylene tibial components when compared to the other components (≥ 12 millimeters).

Using multiple linear regression analysis to remove the confounding effects of age, duration of implantation, we found no statistically significant relationship between the postoperative hip-knee-ankle angle of the knees and the linear or volumetric wear-penetration rate (p = 0.26, and p = 0.32 respectively).

### 2 Decrease of thickness of the implant due to creep or deformation of the polyethylene:

**Fig. (6):**
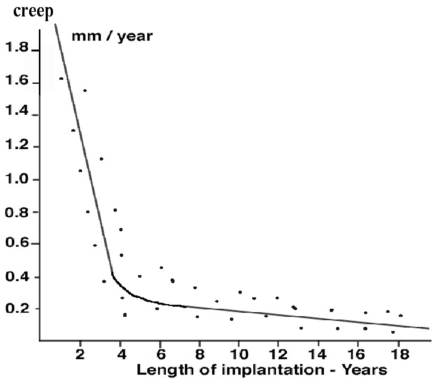
Decrease of thickness of the implant due to creep compared to the duration of implantation.

The amount of decrease in thickness of the implant due to creep and calculated with the gravimetric method was average 1.5 millimeters (range 0.9 to 3.1 millimeters). The linear rate of decrease thickness of the implant due to creep ranged from 0.07 to 1.9 millimeter per year (mean 0.12 millimeter per year). The linear creep rates were higher in the first two years after the implantation compared to the subsequent years but did not seem to be completely stopped after the second year of implantation (Fig. **6**).

We analyzed the influence on creep on the following criteria: patient's age, gender and weight; the thickness of the polyethylene and the alignment of the limb with the hip-knee-ankle. Using the Spearman test, the amount of linear creep showed a positive correlation (p < 0.05) with patient weight (Rs = 0.39), and with increased postoperative varus deformity (Rs = 0.38).

The patient's age and the gender had no significant relation to the amount of creep with the number of cases available (p = 0.46). On the other hand the patient's weight did have a significant relation to the amount of creep. The patients with the greater amounts of creep (greater than 1.5 millimeters) were significantly (p = 0.03) heavier (mean, 96 kilograms; 95 per cent confidence interval 78 to 115) than those (mean, 73 kilograms; 95 per cent confidence interval 52 to 94) with the lower amount of creep (less than 1.5 millimeters).

We examined the thickness of the tibial component and found that the twenty-seven implants with the highest amounts of creep (greater than 1.5 millimeters) had a significantly (p = 0.02) higher percentage (74 per cent; 20/27) of thinner tibial implants (≤ 9 millimeters) than those with the lowest amounts of creep (32 per cent; 9/28). The decrease of thickness due to creep (or deformation) was significantly greater (p < 0.02) in the six thin (six millimeters) implants than in the others.

After adjusting with multiple linear regression for weight, duration of implantation and original thickness of the implant, the mean postoperative varus deformity (mean hip-knee-ankle angle: 166 degrees) of the knees with the highest amount of creep (greater than 1.5 millimeters) was significantly (p = 0.03) greater than the mean postoperative varus deformity (mean hip-knee-ankle angle: 176 degrees) of the knees with the lowest amount of creep (less than, 1.5 millimeters).

### 3 Total linear penetration of the femoral component in the polyethylene:

As the result of creep and true wear the total linear penetration of the femoral condyle into the polyethylene ranged from 0.18 to 2.6 millimeter per year (mean 0.25 millimeter per year). At the time of implantation, as specified in the manufacturers packaging the thickness of the implants ranged from six millimeters to fifteen millimeters. The actual measured thickness of the polyethylene at the thinnest point was from 7.8 to 2.1 millimeter (mean 3.1 millimeters) less than the measurement listed at the time of implantation on the implant packaging. The mean remaining thickness of the polyethylene at revision was only 4.5 millimeters (range 1.2 to 6.3 millimeters) for implants with loosening and the remaining thickness of the polyethylene was average 9.16 millimeters (range 7.2 to 10.7 millimeters) in absence of loosening and the difference was significant between the two groups (p = 0.001).

The mean total linear penetration (2.1 millimeters) in the thirteen knees with absence of loosening was significantly less (p = 0.04) than the mean total linear penetration in the forty-two implants with tibial loosening (mean 3.4 millimeters). After adjusting for age, weight, gender and the thickness of the implant, the mean postoperative varus deformity was also significantly (p = 0.03) less in the thirteen knees without loosening (mean hip-knee-ankle angle: 175 degrees) than in the forty-two knees with loosening (mean hip-knee-ankle angle: 169 degrees). But we did not find that the volume of wear was significantly (p > 0.05) greater for the forty-two implants with loosening than for the thirteen implants without loosening.

We examined also the thickness of the tibial implant and found that the forty-two implants revised for loosening had a significantly (p = 0.001) higher percentage (71 per cent; 30/42) of thinner implants (≤ 9 millimeters) than those (thirteen implants) that were revised for another reason than loosening of the tibial implant (8 per cent; 1/13).

A comparison of age also showed that patients in the group of knees with loosening of the tibial plateau were significantly younger (p < 0.05) than patients in the group of knees revised for another reason than loosening.

## DISCUSSION

The current study evaluated plastic deformation (creep) and true wear of tibial inserts obtained at the time of revision of unicompartmental arthroplasties. Several tibial polyethylene wear mechanisms [3-7] have been described for the knee. However there have been few studies to investigate separately the creep and wear of full polyethylene tibial implants in knee arthroplasties. Recently, Harman *et al*. [8] described a quantitative metrological method to separate creep and wear in retrieved polyethylene implants. Muratoglu [9] used such a method on implants tested on knee simulator.

Linear and volumetric penetration rates due to true wear were negatively correlated with the duration of implantation. When wear rates were plotted against the duration of implantation, there proved to be a higher wear rate in the first years of implantation compared to subsequent years. After the third year of implantation the linear penetration rate due to true wear remained fairly constant or actually decreased. The relationship between decreased rates of wear and increased time in situ, has several implications. It is possible that the deformation and the so-called wearing-in phenomenon occur early on, during which time the incongruities between the femoral component and the tibial polyethylene are worn away at a higher rate. The articulation may then become more congruous, leading to a lower rate of wear.

In the current study, The most useful implants were those not removed for loosening, however they were few in number, and therefore when compared to the others be underpowered. The analysis demonstrated that the postoperative deformity and weight did not have a significant relationship to the rate of wear. However, age had a significant relationship to the rate of wear; the rate of wear being significantly higher in younger patients. This finding is also consistent with abrasive and adhesive mechanisms of wear rather than a fatigue mechanism, since with the former mechanisms the distance travelled by the femoral component within the tibial polyethylene (sliding and rolling distance) would be a more important determinant of wear than the loads imposed on the tibia. It is generally assumed that in heavier patients the knees are subjected to a higher load and that they are therefore more likely to have higher rates of wear. However, this parameter (weight) may not be a good indicator of level of activity or of the number of cycles imposed on the tibia each year. The number of cycles and the sliding distance may be more important determinants of wear than the joint load (postoperative deformity and weight) if abrasion and adhesion are the predominant mechanisms of wear. However heavy patients had increased creep deformation.

**Fig. (7):**
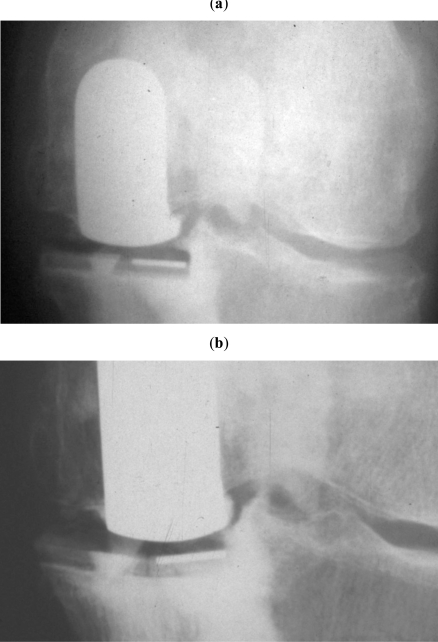
(**a**) radiograph of an implant 6 mm thick just after the operation. (**b**) the same implant at a two year follow-up examination = creep is evident on radiograph. Creep of the polyethylene, but also creep of the cement can be observed on the radiograph of the knee with a postoperative varus deformity (hip knee ankle angle: 168 degrees).

The exact rate of penetrative creep is difficult to predict because its initial component is very rapid and the amount of creep is difficult to quantify. Furthermore, there may be effects of interaction between the creep and wear elements of the penetration as both alter and are influenced by the contact regime of the components. This study demonstrated however that there was an initial creep deformation during the first years of implantation. There is a settling in period during initial loading of the polyethylene bearing surfaces with a higher initial deformity rate in the first years. Creep deformation was significantly higher in heavy patients and in tibial inserts implanted in knees with severe postoperative varus deformity as opposed to those implanted in knees without severe postoperative varus deformity. Linear creep rates were also negatively correlated with implant thickness (Fig. **7**). The explanation is that polyethylene-bearing surfaces undergo plastic deformation, especially in flat and thin designs [10-12]. This theory is supported by the higher amount of creep in implants with malalignment of the knee and in inserts implanted in heavy patients. In these implants it was found that a great part of the decrease in thickness was due to the creep deformation.

In conclusion, our study suggests that surgeons using the Lotus Mk I unicompartmental knee replacement for medial tibiofemoral arthritis should beware of the overweight patient (> 90kg) with a tibial implant of less than 9 mm. There is a risk of increased penetration and decreased thickness of the tibial implant when it is too thin, the knee malaligned, and the patient heavy. All these factors increase creep deformation.

## References

[1] Bartel DL, Bicknell VL, Wright TM (1986). The effect of conformity, thickness and material on stresses in ultra high molecular weight components for total joint replacement. J Bone Joint Surg (Am).

[2] Ly S, Brustein AH (1994). Ultra high molecular weight polyethylene. J Bone Joint Surg (Am).

[3] Bartley RE, Stulberg SD, Robb WJ, Sweeney HJ (1994). Polyethylene wear in unicompartmental knee arthroplasty. Clin Orthop Rel Res.

[4] Blunn GW, Joshi AB, Lilley PA (1992). Polyethylene wear in unicondylar knee prostheses. 106 retrieved Marmor PCA and St Georg tibial components compared. Acta Orthop Scand.

[5] Choon JJL, Rand JA (1993). Revision of failed unicompartmental knee arthroplasty. Clin Orthop.

[6] Lindstrand A, Stenstrom A (1992). Polyethylene wear of the PCA unicompartmental knee. Prospective 5 (4-8) year study of 120 arthrosis knees. Acta Orthop Scand.

[7] Feng EL, Stulberg SD, Wixson RL (1994). Progressive subluxation and polyethylene wear in total knee replacements with flat articular surfaces. Clin Orthop.

[8] Harman MK, Banks SA, Pue E, Hugde WA (2000). Depth and rate of surface deformation or retrieved polyethylene tibial inserts. Trans Orthop Res Soc.

[9] Muratoglu O, Perinchief R, Bradgon CR, O'Connor D, Konrad R, Harris N (2004). Metrology to quantify wear and creep of polyethylene tibial knee inserts. Chir Orthop.

[10] Herzog R, Morscher E (1991). Failures of knee joint prostheses. An analysis of knee prostheses and component revisions, 1980-1987. Orthopade.

[11] Hood RW, Wright TM, Burstein AH (1983). Retrieval analysis of total knee prostheses: a method and its application to 48 total condylar prostheses. J Biomed Mater Res.

[12] Wasielewski RC, Galante JO, Leighty RM, Natarajan RN, Rosenberg AG (1994). Wear patterns on retrieved polyethylene tibial inserts and their relasionship to technical considerations during total knee arthroplasty. Clin Orthop.

